# Changes of radiological examination volumes over the course of the COVID-19 pandemic: a comprehensive analysis of the different waves of infection

**DOI:** 10.1186/s13244-022-01181-z

**Published:** 2022-03-07

**Authors:** Florian Nima Fleckenstein, Tazio Maleitzke, Georg Böning, Vinzent Kahl, Alexandra Petukhova-Greenstein, Ahmet Said Kucukkaya, Bernhard Gebauer, Bernd Hamm, Annette Aigner

**Affiliations:** 1 Department of Diagnostic and Interventional Radiology, Charité – Universitätsmedizin Berlin, corporate member of Freie Universität Berlin and Humboldt-Universität Zu Berlin, Augustenburger Platz 1, Berlin, Germany; 2 BIH Charité Clinician Scientist Program, Berlin Institute of Health at Charité – Universitätsmedizin Berlin, BIH Biomedical Innovation Academy, Berlin, Germany; 3 Center for Musculoskeletal Surgery, Charité – Universitätsmedizin Berlin, corporate member of Freie Universität Berlin and Humboldt-Universität Zu Berlin, Berlin, Germany; 4 Julius Wolff Institute, Berlin Institute of Health at Charité – Universitätsmedizin Berlin, Berlin, Germany; 5 Institute of Biometry and Clinical Epidemiology, Charité – Universitätsmedizin Berlin, corporate member of Freie Universität Berlin and Humboldt-Universität Zu Berlin, Berlin, Germany

**Keywords:** SARS-CoV-2, Lockdown, Public Health, Crisis, Healthcare

## Abstract

**Objectives:**

Data from radiological departments provide important information on overall quantities of medical care provided. With this study we used a comprehensive analysis of radiological examinations as a surrogate marker to quantify the effect of the different COVID-19 waves on medical care provided.

**Methods:**

Radiological examination volumes during the different waves of infection were compared among each other as well as to time-matched control periods from pre-pandemic years using a locally weighted scatterplot smoothing as well as negative binominal regression models.

**Results:**

A total of 1,321,119 radiological examinations were analyzed. Examination volumes were reduced by about 10% over the whole study period (IRR = 0.90; 95% CI 0.89–0.92), with a focus on acute medical care (0.84; 0.83–0.85) and outpatients (0.93: 0.90–0.97). When compared to wave 1, examination volumes were about 17% higher during wave 2 (1.17; 1.10–1.25), and 33% higher in wave 3 of the pandemic (1.33; 1.24–1.42).

**Conclusions:**

This study shows the severe effect of COVID-19 pandemic and related shutdown measures on overall provided medical care as measured by radiological examinations. When compared, the decrease of medical care was more pronounced in the earlier waves of the pandemic.

**Supplementary Information:**

The online version contains supplementary material available at 10.1186/s13244-022-01181-z.

## Key points

- Over the course of four waves of the COVID-19 pandemic in the years 2020/21, the number of radiological examinations decreased significantly as compared to baseline data from 2018/19.
- The decrease was more pronounced in the field of acute medical care than in outpatient settings.
- When compared to wave 1, examination volumes were about 17% higher during wave 2, and 33% higher in wave 3 of the pandemic, indicating a lower impact of the two latter waves on provided medical care.

## Introduction

As a result of the rapid worldwide spread of the novel coronavirus disease 2019 (COVID-19) [1] caused by the severe acute respiratory syndrome coronavirus 2 (SARS-CoV-2), governments all over the world implemented overarching measures to contain the pandemic and to prevent a failure of healthcare infrastructures [2–4].

Although quarantine laws, travel restrictions, as well as an effective and inclusive vaccination policy are believed to play an important role in relieving pressure on healthcare organizations, the average age of a community seems to be the most crucial factor influencing how many hospital admissions are registered [4]. To be prepared for an increased need for intensive care unit (ICU) capacities, major adjustments in healthcare infrastructure were undertaken for the care of COVID-19 just as for non-COVID-19 patients. On March 16th, 2020, the German federal government passed a law, known as the “COVID-19-Krankenhausentlastungsgesetz” to compensate hospitals and other healthcare facilities financially for the reduction in elective care and for hospitals which allocated ICU beds to COVID-19 patients [5]. Starting on April 20th, 2020, the first relaxations of these measures were decided, including a resumption of elective healthcare [6]. During the fall of 2020 COVID-19 cases began to rise again and lockdown measures were reinstated starting on November 11th, 2020 with the beginning of the second wave of the pandemic through January 29th, 2021 [7]. After a short time of stabilization on March 3^rd^, 2021, the federal government implemented the so-called emergency brake (“Notbremse”) [6]. This emergency plan effectively standardized the pandemic response across all of Germany. COVID-19 cases rose into a third wave in Germany in April 2021, and elective hospital treatments were postponed starting from April 21th, 2021, especially as ICUs were nearing critical capacities. With these measures being successful and incidences decreasing, it was officially announced that after May 31st, 2021, hospitals would no longer need to reserve capacities for COVID-19 patients, signaling a return to regular care [6].

While the fourth wave of the pandemic is ongoing and lockdown measures are being reinstalled, profound data about the early waves of the pandemic are only now beginning to be published [8–12]. This leads hospitals and healthcare providers into an unknown future given the increasing clinical and economic pressure. Yet, the challenge to analyze and understand the changes in the system remains unaddressed. There is an urgent need not only to guide hospitals through a time of organizational and financial strains but also to create resilient and prepared healthcare structures for future pandemic situations.

In this context, data from radiological departments, as a cross-disciplinary assessment, can provide important information on overall quantities of medical care provided for COVID-19 as well as non-COVID-19 patients [11, 13].

This study expands on a recent publication from our study group [11] and analyzes comprehensive volumes of radiological examinations during the second and third waves of the pandemic as a surrogate marker for the quantity of medical care provided. Changes are compared to inter- and intra-year control periods, especially those which were affected during waves of infection.

## Methods

This study was approved by the Local Ethics Committee and was conducted following the Declaration of Helsinki. We conducted a comprehensive, retrospective study including all three campuses of the Charité Universitätsmedizin Berlin, Germany. All involved centers are maximum-care hospitals. All data was extracted anonymously from the department’s radiological database for the study periods.

### Study periods

Data on radiology examinations was obtained from January 1st, 2020, through July 15th, 2021, and as a reference period also from January 1st, 2018, through July 15th, 2019. To analyze the effect of the COVID-19 pandemic and the measures which were implemented, different study periods were defined:

1. First wave shutdown period (wave 1) from March 16th until April 19th, 2020
2. Second wave shutdown period (wave 2) from December 11th, 2020, to January 29th, 2021
3. Hospital shutdown period for the third wave (wave 3) from April 21st, 2021, to May 31st, 2021
4. Control periods in 2018/2019 for all aforementioned periods

### Data preparation

Clinically irrelevant imaging procedures were excluded from the analysis, comprising (i) image duplicates, (ii) external images which were imported into our database, and (iii) test images. Each radiological examination was then stratified in regard to modality and medical specialty from which it was referred. Examinations from out-patient centers, ICUs, and emergency rooms (ER) were registered as such. Furthermore, all radiological examinations linked to a SARS-CoV-2 infection were registered by automatically searching all patient reports for the following terms: “COVID”, “SARS-CoV-2”, “C-ARDS” and “Corona”. Of note, examinations were aggregated daily and for patient-based analyses, each patient was only counted once per study period.

### Statistical analysis

Absolute and relative frequencies are derived for categorical variables, median and interquartile range (IQR), or mean and standard deviation (SD) for continuous variables. Absolute numbers of radiological examinations are displayed over the full study period 2020/2021. Daily numbers are plotted over the same period, just as the control period 2018/2019, using a locally weighted scatterplot smoothing (LOESS) estimate to visualize global trends over time. These analyses were also stratified regarding regular shifts and weekends/holidays/night shifts, just as emergency vs. outpatients. Additionally, time trends in median age and the ratio of men in comparison to women are displayed, just as the mean number of examinations per day by study periods, along with 95% confidence intervals (CI).

To assess inter- and intra-year differences in the daily volumes of examinations, unstratified and stratified negative binomial regression models were performed, accounting for effects of weekdays, weekends, and holidays.

All statistical analyses were performed in R [14], with additional R packages [15, 16].

## Results

**Fig. 1:**
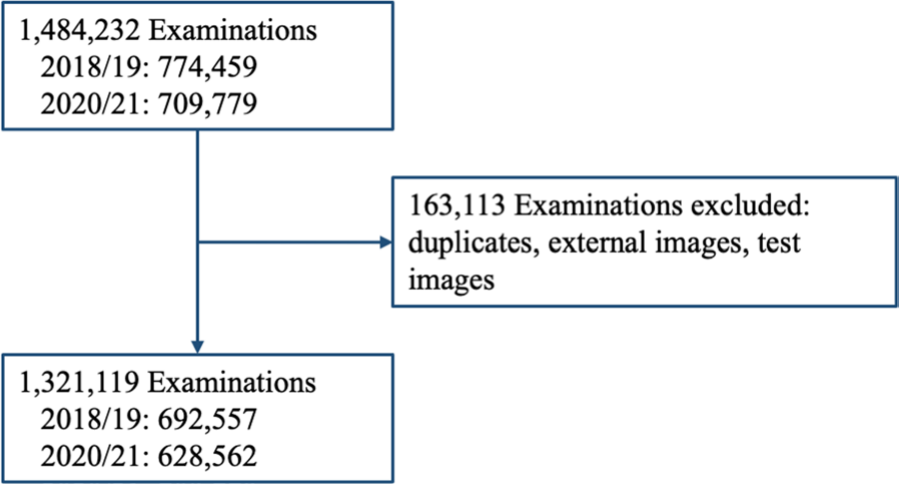
Flowchart of all radiological examinations included in the final analysis. A total of 163,113 cases were excluded due to clinically non-relevant imaging

After exclusion of clinically irrelevant imaging, this study included a total of 1,321,119 radiological examinations into the final analysis consisting of 692,562 examinations based on 211,839 patients for the control period in 2018/19 and 628,572 examinations on 186,660 patients in 2020/21 (Fig. 1). The median number of examinations per patient was 2 (IQR 1–5). Between 2018/19 and 202/21, the proportion of males and females was similar, with a median age of 54 (IQR 34–70) in 2018/19, and of 55 (IQR 34–70) in 2020/21 (Table 1).

**Table 1:** Number of examinations and patients per year (January 1st–July 15th, 2021 with reference years 2018/19) and demographic information of patients

	2018/19	2020/21	Total
Total number of exams	692,557	628,562	1,321,119
**Campus**			
Mitte	217,503 (31.4%)	195,350 (31.1%)	412,853 (31.3%)
Benjamin Franklin	187,582 (27.1%)	169,632 (27.0%)	357,214 (27.0%)
Virchow-Klinikum	287,472 (41.5%)	263,580 (41.9%)	551,052 (41.7%)
Total number of patients	211,839	186,660	398,499
Median examinations per patient (IQR)	2.0 (1.0–5.0)	2.0 (1.0–5.0)	2.0 (1.0–5.0)
**Sex**			
Women	104,519 (49.3%)	90,988 (48.7%)	195,507 (49.1%)
Men	106,519 (50.3%)	94,756 (50.8%)	201,275 (50.5%)
Diverse	2 (0.0%)	3 (0.0%)	5 (0.0%)
Unknown	799 (0.4%)	913 (0.5%)	1712 (0.4%)
**Age**			
Median (IQR)	54.0 (34.0, 70.0)	55.0 (34.0, 70.0)	55.0 (34.0, 70.0)

IQR, interquartile range

Distinguished by the three COVID-19 waves of infection in 2020/21, 26,967 radiological examinations were carried out in the first, 46,300 in the second, and 42,124 in the third wave, based on 22,876; 26,495; and 23,724 patients, respectively. There was no relevant difference between the waves regarding patients’ gender, yet median age was slightly lower in wave 2 (median = 55, IQR 34–71) and even lower during wave 3 (median = 53, IQR 32–70), compared to wave 1 (median = 56, IQR 36–71, Table 2 as well as Additional file 1: Figs. S1 and S2).

**Table 2:** Absolute number of examinations and average examinations per day during each wave of the COVID-19 pandemic

	Wave 1 (35 days)	Wave 2 (50 days)	Wave 3 (41 days)
2020/21: Total number of exams (mean per day)	26,967 (770.5)	46,300 (926.0)	42,124 (1027.4)
2018/19: Total number of exams (mean per day)	42,107 (1203.1)	57,674 (1153.5)	50,718 (1237.0)
**Timepoint of Acquisition**			
Regular working hours	21,642 (80.3%)	37,783 (81.6%)	33,796 (80.2%)
Weekends / holidays / night shifts	5325 (19.7%)	8517 (18.4%)	8328 (19.8%)
**Emergency rooms and out-patient centers**			
Out-patient centers	5114 (19.0%)	11,403 (24.6%)	11,224 (26.6%)
ER	4876 (18.1%)	7844 (16.9%)	7390 (17.5%)
Other	16,977 (63.0%)	27,053 (58.4%)	23,510 (55.8%)
**Modality**			
Sonography	2305 (8.5%)	4757 (10.3%)	5058 (12.0%)
X-Ray	8239 (30.6%)	14,467 (31.2%)	14,544 (34.5%)
MRI	4039 (15.0%)	6595 (14.2%)	5753 (13.7%)
CT	7022 (26.0%)	11,890 (25.7%)	9796 (23.3%)
Other	5362 (19.9%)	8591 (18.6%)	6973 (16.6%)

Wave 1: 16-03-2020–19-04-2020; Wave 2: 11-12-2020–29-01-2021; Wave 3: 21-04-2021–31-05-2021

ER, emergency room; MRI, magnetic resonance imaging; CT, computed tomography

**Fig. 2:**
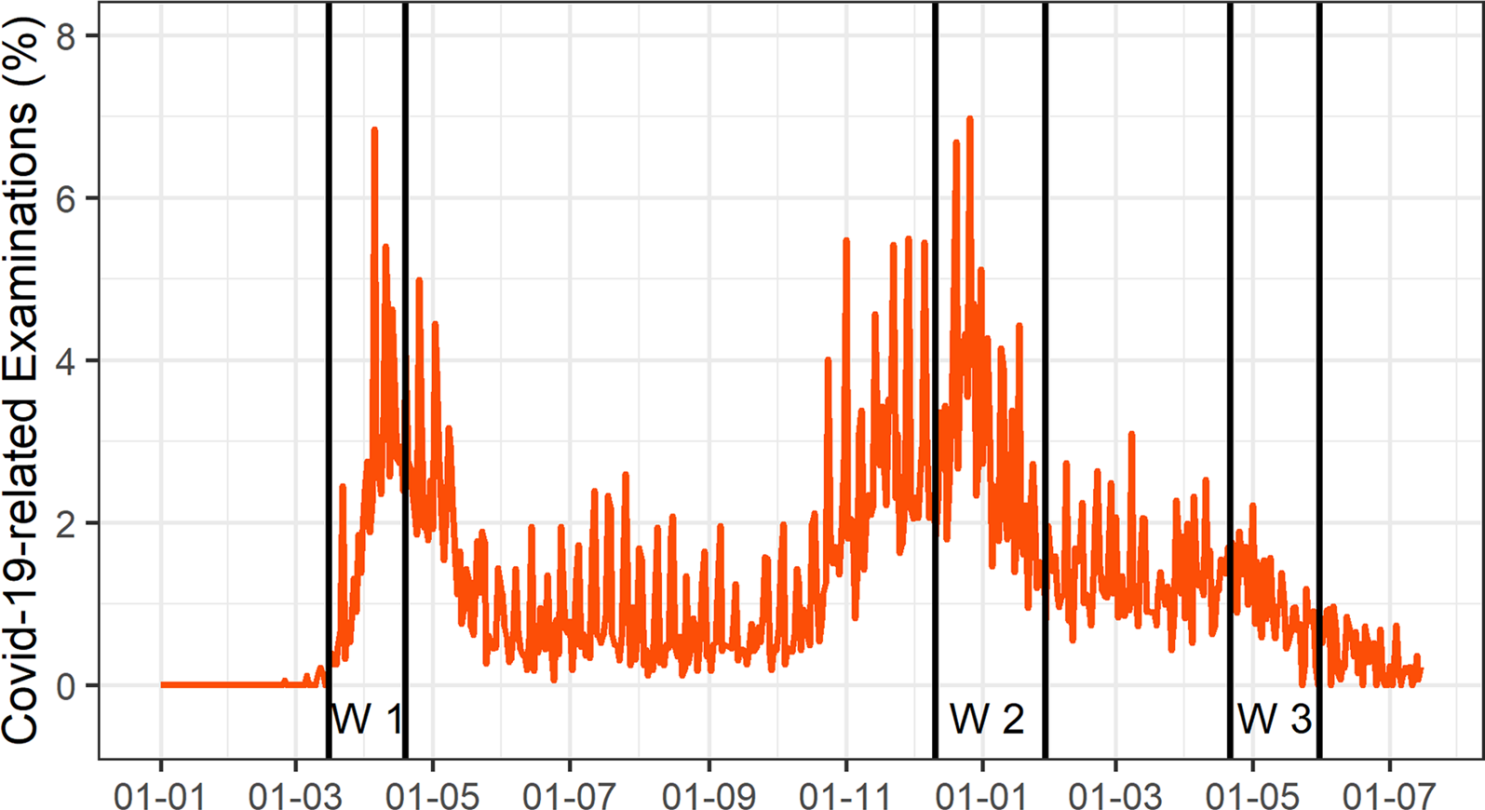
SARS-Cov-2-related radiological examinations. Percentage of examinations reported due to a suspected or confirmed SARS-CoV-2 infection among all performed examinations. Indicated are the following periods: Wave 1: 16-03-2020–19-04-2020; Wave 2: 11-12-2020–29-01-2021; Wave 3: 21-04-2021–31-05-2021

During 2020/21, 6,324 examinations were carried out as reported due to a suspected or confirmed SARS-CoV-2 infection, out of which 513 were registered in the first wave, 1145 in the second, and 431 in the third wave (Fig. 2).

**Fig. 3:**
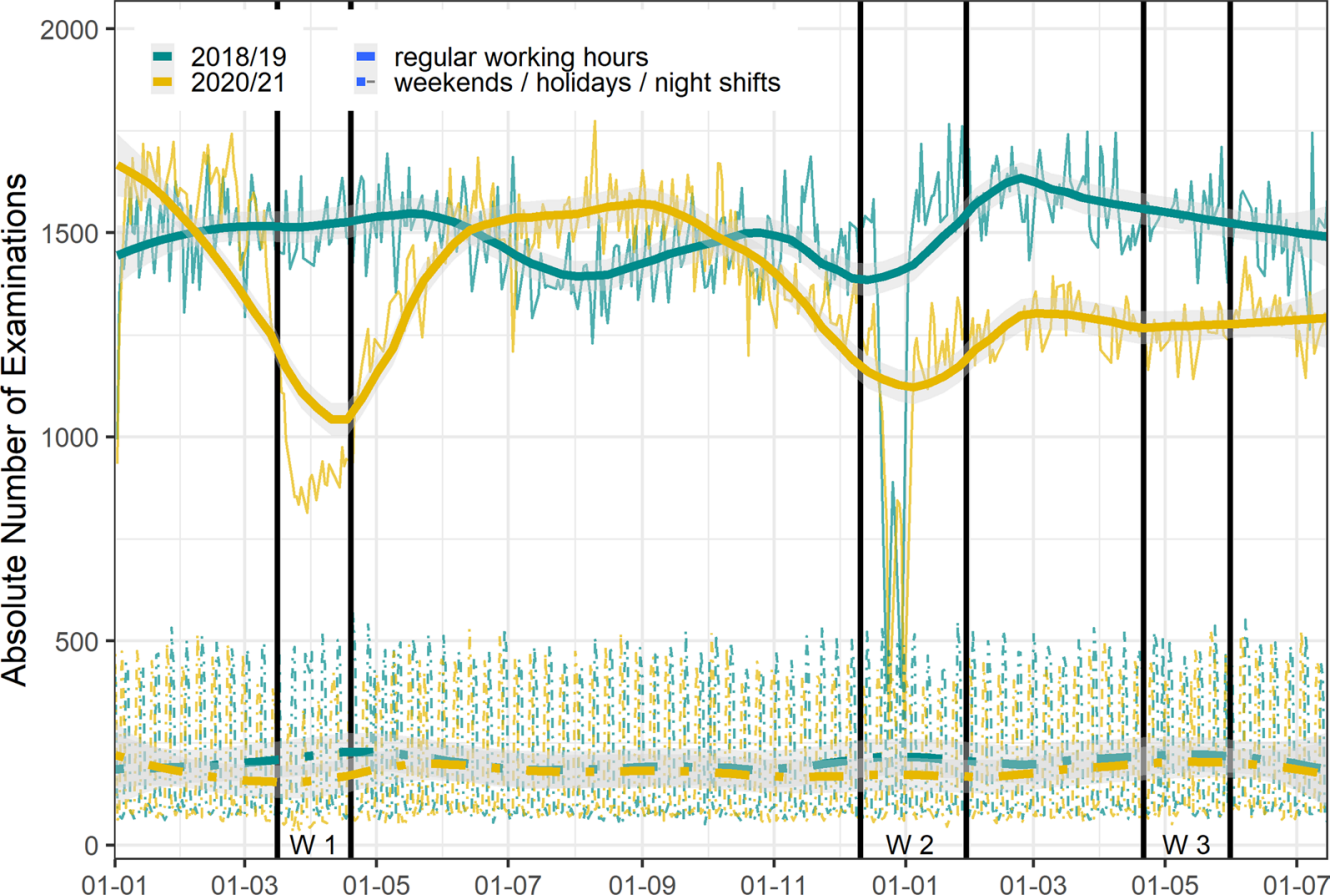
Changes of absolute number of examinations over time and with LOESS estimate by regular working hours and night shifts / weekends / holidays (January 1st, 2020, through July 15th, 2021). Broad line shows the smoothened LOESS estimate, the fine line shows absolute numbers of examinations, respectively. Grey areas mark 95% CIs. Wave 1: 16-03-2020–19-04-2020; Wave 2: 11-12-2020–29-01-2021; Wave 3: 21-04-2021–31-05-2021; LOESS: locally weighted scatterplot smoothing; CI, confidence interval

**Fig. 4:**
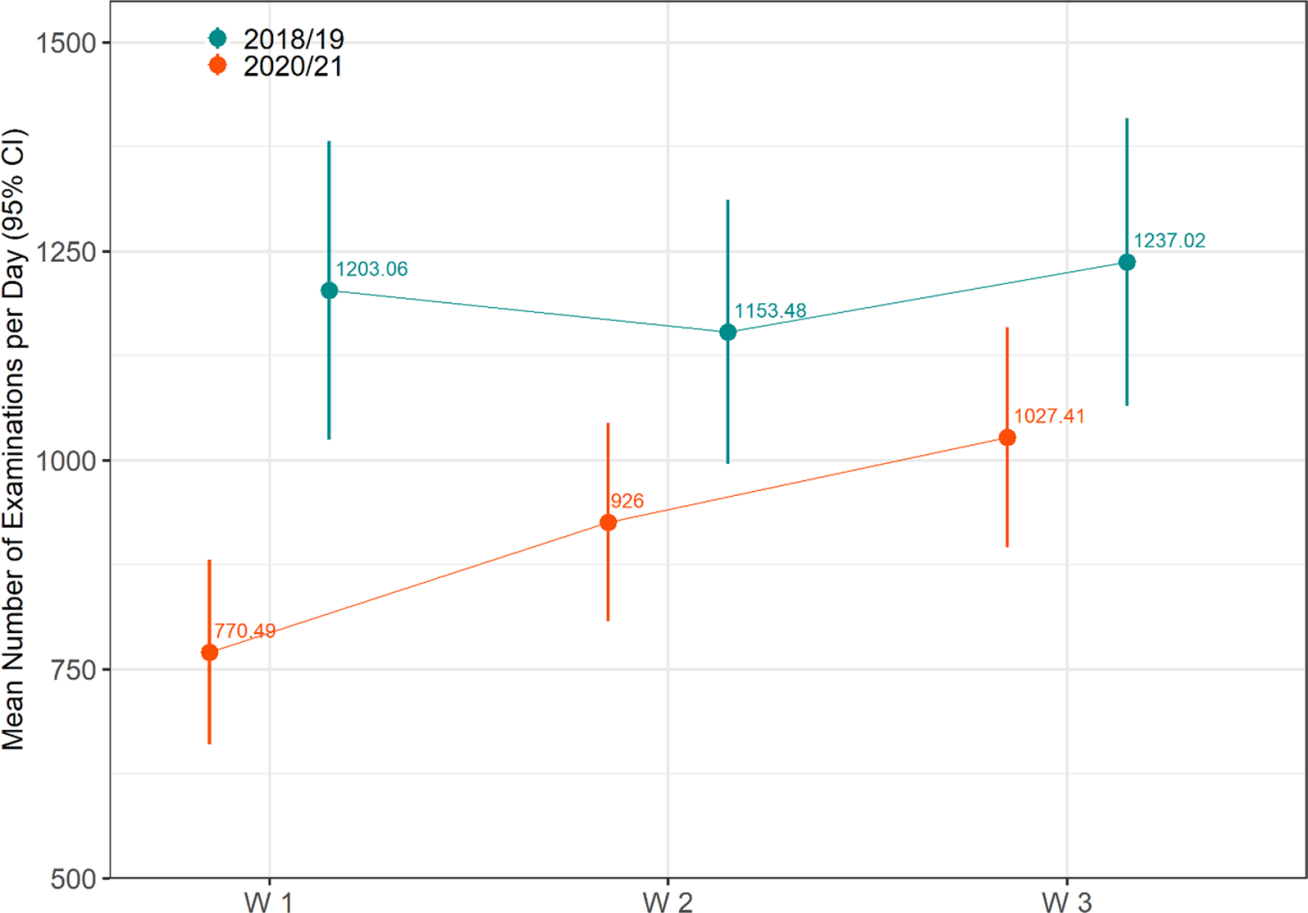
Average Number of Examinations per Day for each Study Period for 2020/21, compared to the respective periods in 2018/19, along with 95% CI. Definition of study periods: Wave 1: 16-03-2020–19-04-2020; Wave 2: 11-12-2020–29-01-2021; Wave 3: 21-04-2021–31-05-2021; CI, confidence interval

In February 2020, a reduction of examination volumes is registered, with the lowest number of examinations in April 2020. Following an increase after the first wave, the numbers started to decrease again beginning of November 2020, with a minimum beginning of January 2021. In February and March 2021, examination volumes rose again, but stagnated at a lower level than in the control year 2018/19, without any relevant impact of the third wave (Fig. 3). Differentiating between regular work hours and night shifts/weekends, we see that the dip in examination numbers in wave 1 was only due to the decrease in examinations during the regular shifts, similarly for the second wave, where the small dip can rather be explained by the vacation days around New Year’s Eve (Fig. 3). The mean number of observations per day was considerably lower in all three waves in 2020/21 compared to the same periods in 2018/19, this was most pronounced for wave 1, followed by waves 2 and 3 (Fig. 4).

**Fig. 5:**
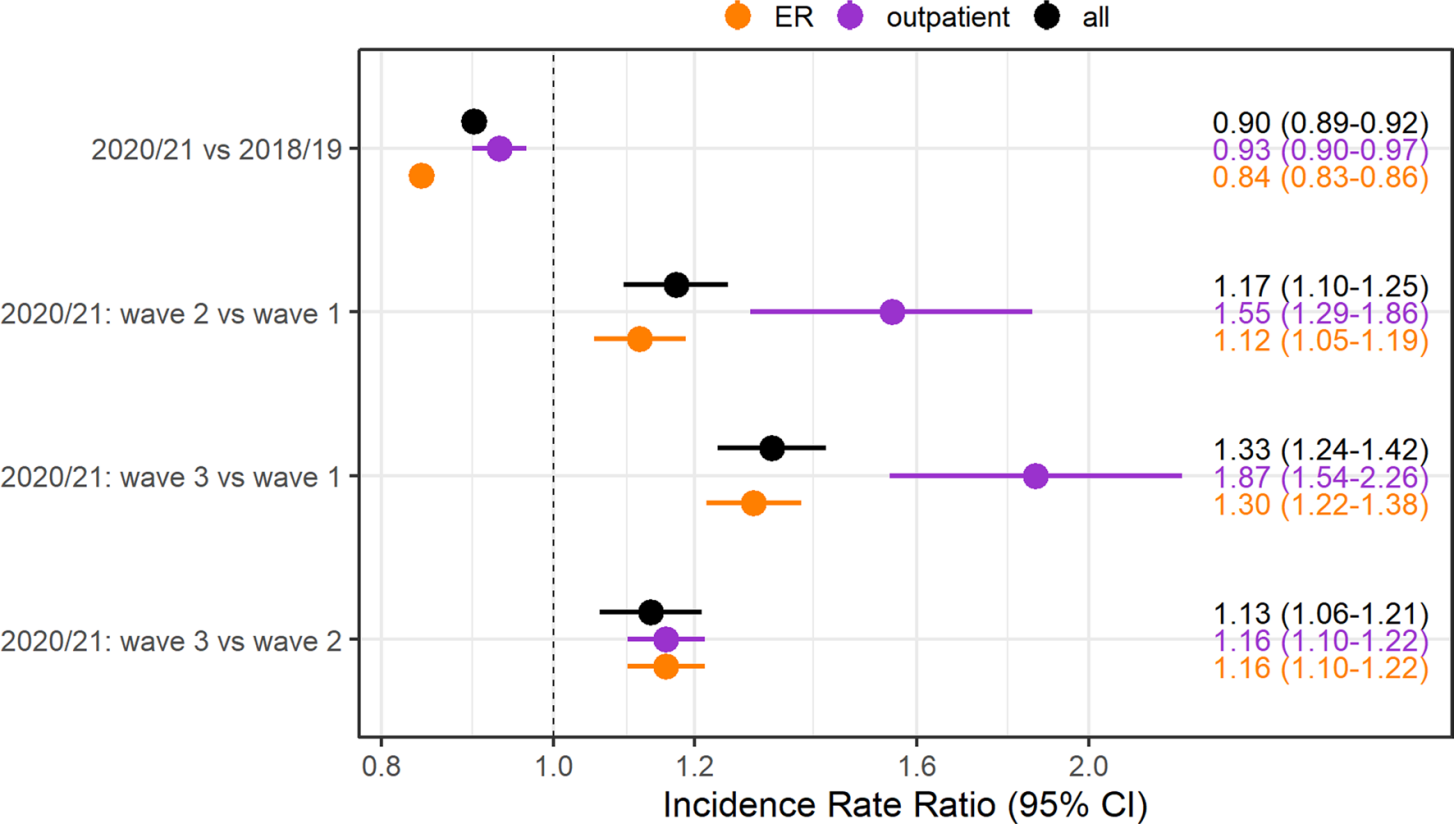
Incidence Rate Ratios unstratified and stratified by ER and outpatients for inter- and intra-year comparison of the study periods, along with 95% CI, derived from the negative binominal regression. The number of examinations was reduced by about 10% over the whole study. When compared to wave 1, examination volumes were about 17% higher during wave 2, indicating a lower impact of the two latter waves on examination volumes. The impact was even lower when only analyzing examinations attributed to outpatient visits, where 55% more examinations were registered in wave 2 and 87% in wave 3 most likely due to postponed examinations from wave 1. (ER, Emergency rooms; CI, confidence intervals)

The descriptive findings were validated with negative binomial regression models adjusting for the effect of weekdays and holidays, with the focus on the comparison between the waves. The number of examinations was reduced by about 10% over the whole study period (IRR = 0.90; 95% CI 0.89–0.92), which was more pronounced in the ER (0.84; 0.83–0.85) than in the outpatient setting (0.93: 0.90–0.97). When compared to wave 1, examination volumes were about 17% higher during wave 2 (1.17; 1.10–1.25), and 33% higher in wave 3 of the pandemic (1.33; 1.24–1.42), indicating a lower impact of the two latter waves on examination volumes. This was even more pronounced when only analyzing examinations attributed to outpatient visits, where 55% more examinations were registered in wave 2 (1.55; 1.29–1.86) and 87% in wave 3 (1.87; 1.54–2.26) when compared to wave 1 (Fig. 5). Additional analyses stratified by regular working hours compared to night shifts, weekends or holidays, show the same trend of higher examination volumes in latter waves of infection (Additional file 1: Fig. S3).

## Discussion

This study demonstrated (i) a considerable decrease in medical care as measured by radiological examination volumes over the course of the COVID-19 pandemic as compared to reference data from pre-pandemic years. Further, it showed that (ii) the decrease was more pronounced during wave 2 when compared to wave 3 where examination volumes were already at a comparatively low level.

The COVID-19 pandemic and the corresponding countermeasures implemented by governments around the world will inevitably continue to have a substantial impact on healthcare providers and patients all over the world [4, 6, 11, 17]. In this context, healthcare providers and relevant administrative players aim to not only care for all patients infected with SARS-CoV-2 but also to minimize the negative impact of the pandemic on patients with conditions other than COVID-19.

A study which has recently been published suggest a significant decrease in all cancer-related patient encounters as a result of the pandemic. The study reports decreases of cancer-related patient encounters of -50% and even -90% for cancer screening procedures such as breast- or colorectal cancer screenings [18]. Other studies showed a significant reduction in trauma-related cases in ER departments [19, 20], as well as a decreased number of admissions for emergencies such as coronary artery disease and cerebrovascular events [21, 22]. A study from a large level I trauma center in Germany found that incidence proportions of traffic-related accidents remained almost constant over the course of the first wave of the pandemic. Yet, the reported date shows a significant reduction of patient contact for less severe conditions such as fractures or infections [20]. Why patients with life-threatening conditions were not seeking medical help provided by ED services remains speculative but our data indicates that the patients with less-severe conditions might have required medical care at a later time. In this context, providing enough capacities for the treatment of acute conditions other than COVID-19 such as acute myocardial infarction and strokes will remain challenging while triage guidelines are being discussed publicly and the German federal constitutional court is summoning the government to determine clear rules [23]. In a previous publication of our study group we also used comprehensive data of radiological examinations as a surrogate marker for provided medical care in the first wave of the pandemic [11]. There we found a significant decrease of 41.1% in the total amount of examinations per day during the first shutdown period in comparison to baseline control years. While multiple clinical disciplines faced a decrease of up to 60%, the study also showed that fields of acute care such as emergency departments were not as strongly affected by the pandemic as other fields of healthcare. Another large study recently published by a group from Germany analyzed the impact of the COVID pandemic on examination volumes using data of the first and second wave from multiple centers [12]. The reported results mostly confirm our results for wave 1 and 2 with a marked difference in examination volumes between both waves. Our results show a clear overshoot in numbers of radiological examinations most likely due to postponed appointments. This generally confirms the prediction model on additional future workload reported previously by our group. In comparison to the data from the first wave we found a less pronounced decrease of radiological examinations during the second and third waves of the pandemic. Between the first and the second wave, the examination volume was higher than during the comparable control timeframes before the pandemic. Interestingly, the effect of the third wave was also significantly less pronounced when compared to the second wave. The reason for this might be the significantly lower levels of examinations carried out before the third wave already. The aftermath of the third wave came into effect while the second wave was still ongoing.

Besides ensuring acute care, similarly important challenges lie ahead of the healthcare system. Our data could show, that during the first wave of the pandemic, examinations of patients with chronic and oncological conditions were postponed on a big scale. This very vulnerable patient cohort is now at risk to suffer the most from further cuts in elective medical care. This backlog of postponed medical care must be further addressed with an expansion of diagnostic and therapeutic capacities.

Interestingly, in depth analyses of our data show that healthcare providers started adapting to the situation over the course of the pandemic. When focusing our analysis on examinations performed in an outpatient setting, we could show a tremendous rise of up to 87% in wave 3 compared to wave 1, most likely due to postponed examinations from wave 1 as well as changes in prioritization guidelines. We believe that besides postponed medical care, healthcare providers might also have shifted workflows towards providing care in outpatient settings. This might be safer in regard to hygienic considerations, preserve resources such as bed occupancies and have a positive economic side effect. This redistribution of medical resources and workforce is crucial, especially while the pandemic continues with record breaking incidences all over Europe. While government officials start realizing the long-lasting effect of this pandemic situation diagnostic and therapeutic workflows need to be further adapted.

Our comprehensive data analysis demonstrates in detail how medical institutions have been affected by the different waves of the pandemic. Hence, we provide profound data for an effective redistribution of resources within hospitals and may support informed decision-making in the preparation of national healthcare systems for the course of the COVID-19 spread or other future pandemics.

This study is limited by its retrospective character, using only data from one university hospital in Germany. Yet, the herein presented numbers were collected from all three maximum-care campuses the Charité Berlin consists of, each as big as an average university hospital. Second, the course of the COVID-19 pandemic in Germany might differ from other countries, therefore conclusions have to be interpreted with caution. Lastly, in this study we used radiological examinations as a surrogate marker for medical care and the collected data might not reflect actual levels of medical care provided. However, with over 1.3 million examinations evaluated, we strongly believe that our analysis is robust and a reliable marker for the quantity of provided medical care.

In summary, we conclude that the second and third waves of the COVID-19 pandemic and related shutdown measures caused a marked decrease in overall provided medical care as measured by radiological examinations. When compared, the decrease of medical care was more pronounced in the earlier waves of the pandemic with an overall major negative impact on examination volumes.

## Supplementary Information

**Additional file 1:** Supplementary files showing the development of median age and the ratio men/women over the study period. Furthermore, Additional binominal regression analyses stratified by regular working hours compared to night shifts, weekends or holidays, confirm the trend of higher examination volumes in latter infection waves.

## Data Availability

The data presented in this study are available on request from the corresponding author. The data are not publicly available due to ethical restrictions.

## Abbreviations

CI: Confidence interval
COVID-19: Novel coronavirus disease
ICU: Intensive care unit
IQR: Interquartile range
IRR: Incidence rate ratio
LOESS: Locally weighted scatterplot smoothing
SARS-CoV-2: Severe acute respiratory syndrome coronavirus 2
SD: Standard deviation
