# Supplementary material for: Changes of radiological examination volumes over the course of the COVID-19 pandemic: a comprehensive analysis of the different waves of infection
Source: Insights Imaging. 2022 Mar 7;13:41. doi: 10.1186/s13244-022-01181-z (PMC8899795; doi:10.1186/s13244-022-01181-z)
Supplement: Supplementary file 1 — Additional file 1: Supplementary files showing the development of median age and the ratio men/women over the study period. Furthermore, Additional binominal regression analyses stratified by regular working hours compared to night shifts, weekends or holidays, confirm the trend of higher examination volumes in latter infection waves. [file 13244_2022_1181_MOESM1_ESM.docx]

**ELECTRONIC SUPPLEMENTARY MATERIAL**

**Changes of radiological examination volumes over the course of the COVID-19 pandemic. Analyzing the waves.**

**Figure S1: LOESS estimate for the development of median age over the study period, along with 95% CI.** Median age was higher in the first two waves of the pandemic when compared to previous years.

Wave 1: 16-03-2020 – 19-04-2020; Wave 2: 11-12-2020 – 29-01-2021; Wave 3: 21-04-2021 – 31-05-2021; CI: confidence interval; LOESS: locally weighted scatterplot smoothing.
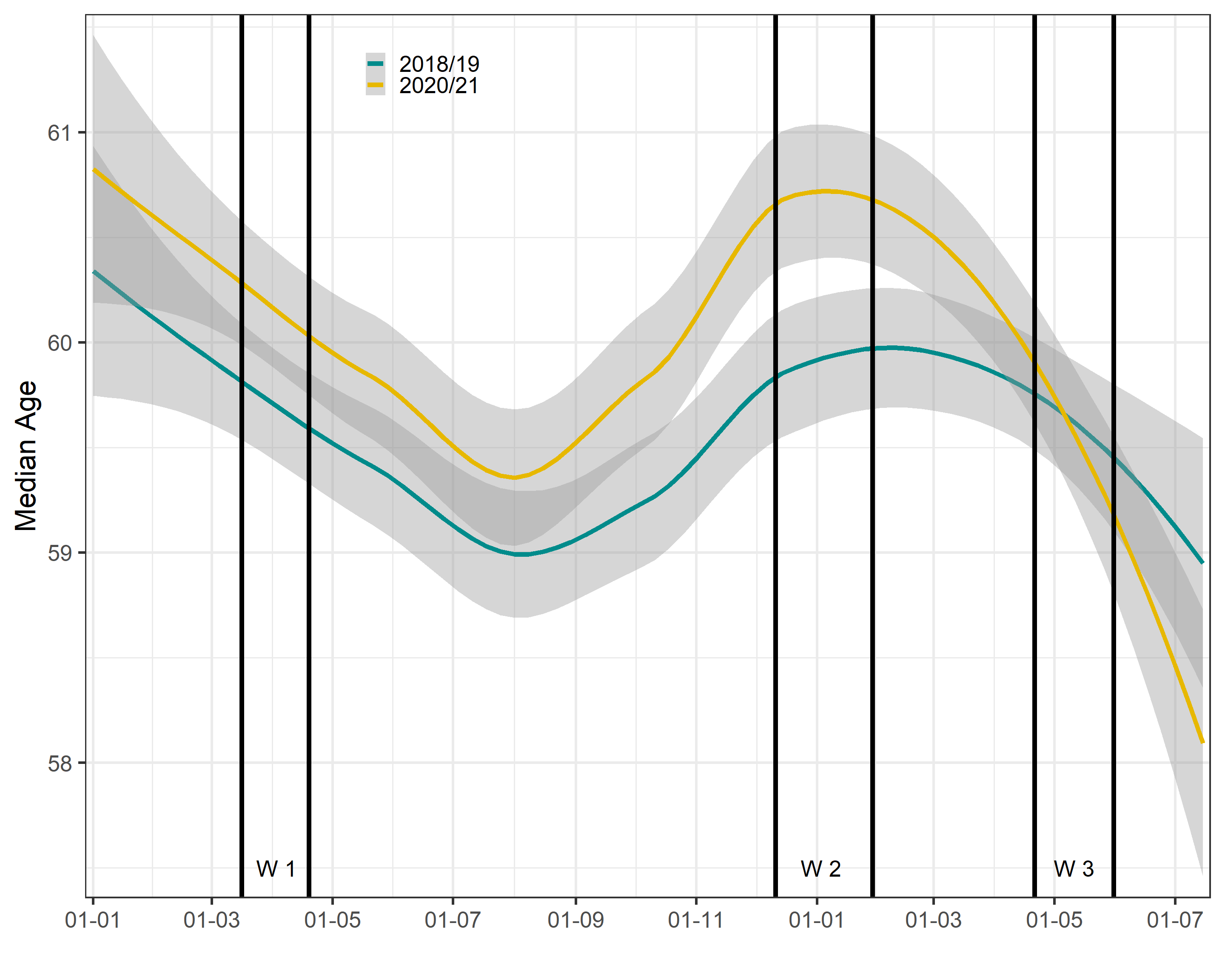


**Figure S2: LOESS estimate for the development of the ratio men/women over the study period, along with 95% CI.** It shows a higher ratio during all waves as compared to the inter-year control period.

Wave 1: 16-03-2020 – 19-04-2020; Wave 2: 11-12-2020 – 29-01-2021; Wave 3: 21-04-2021 – 31-05-2021; CI: confidence interval; LOESS: locally weighted scatterplot smoothing.
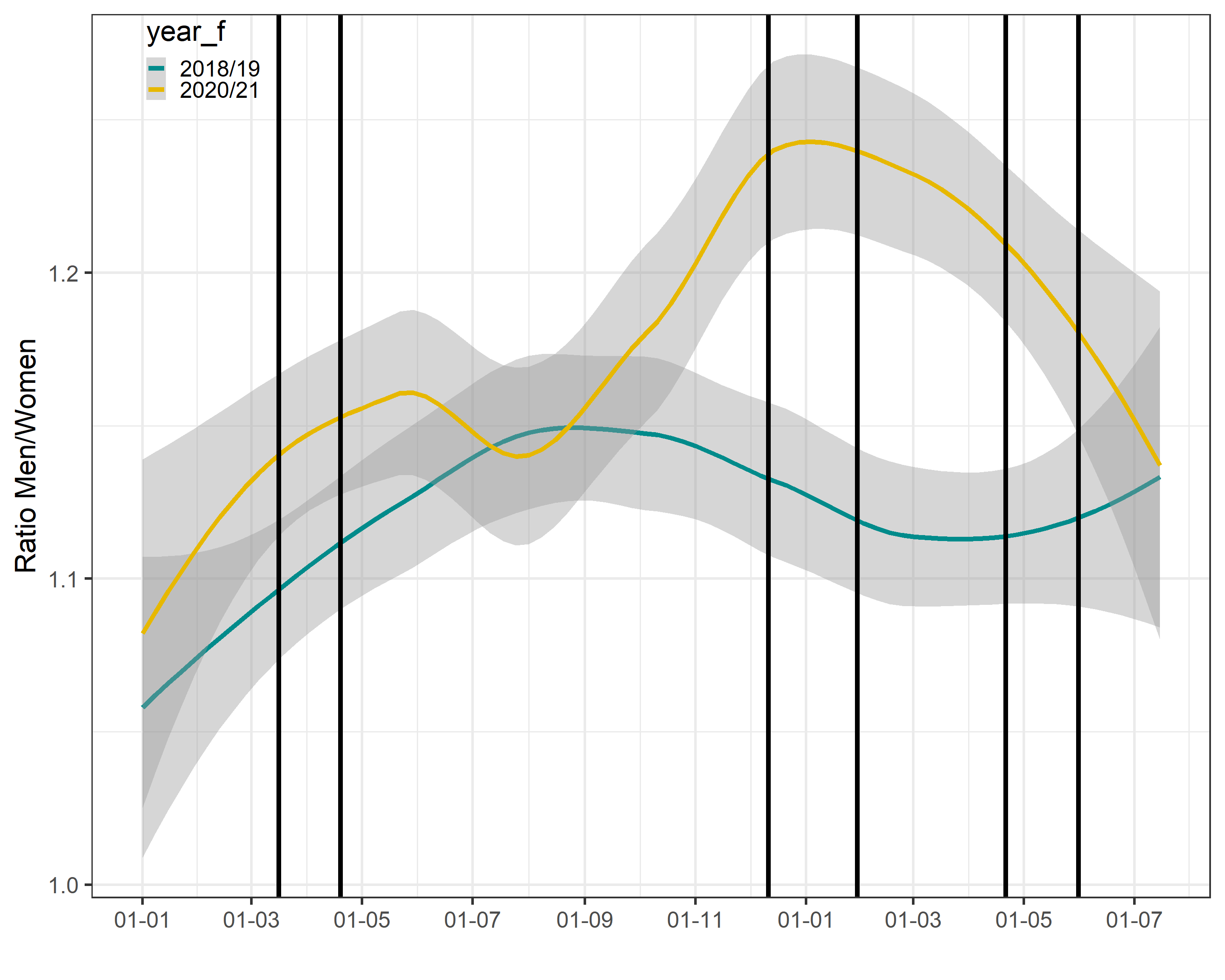


**Figure S3: Incidence Rate Ratios stratified by regular working hours vs. night shift / weekend / holiday for inter- and intra-year comparison of the study periods, along with 95% CI, derived from the negative binominal regression.** Additional analyses stratified by regular working hours compared to night shifts, weekends or holidays, shows confirm the trend of higher examination volumes in latter infection waves.

(CI: confidence intervals).

**
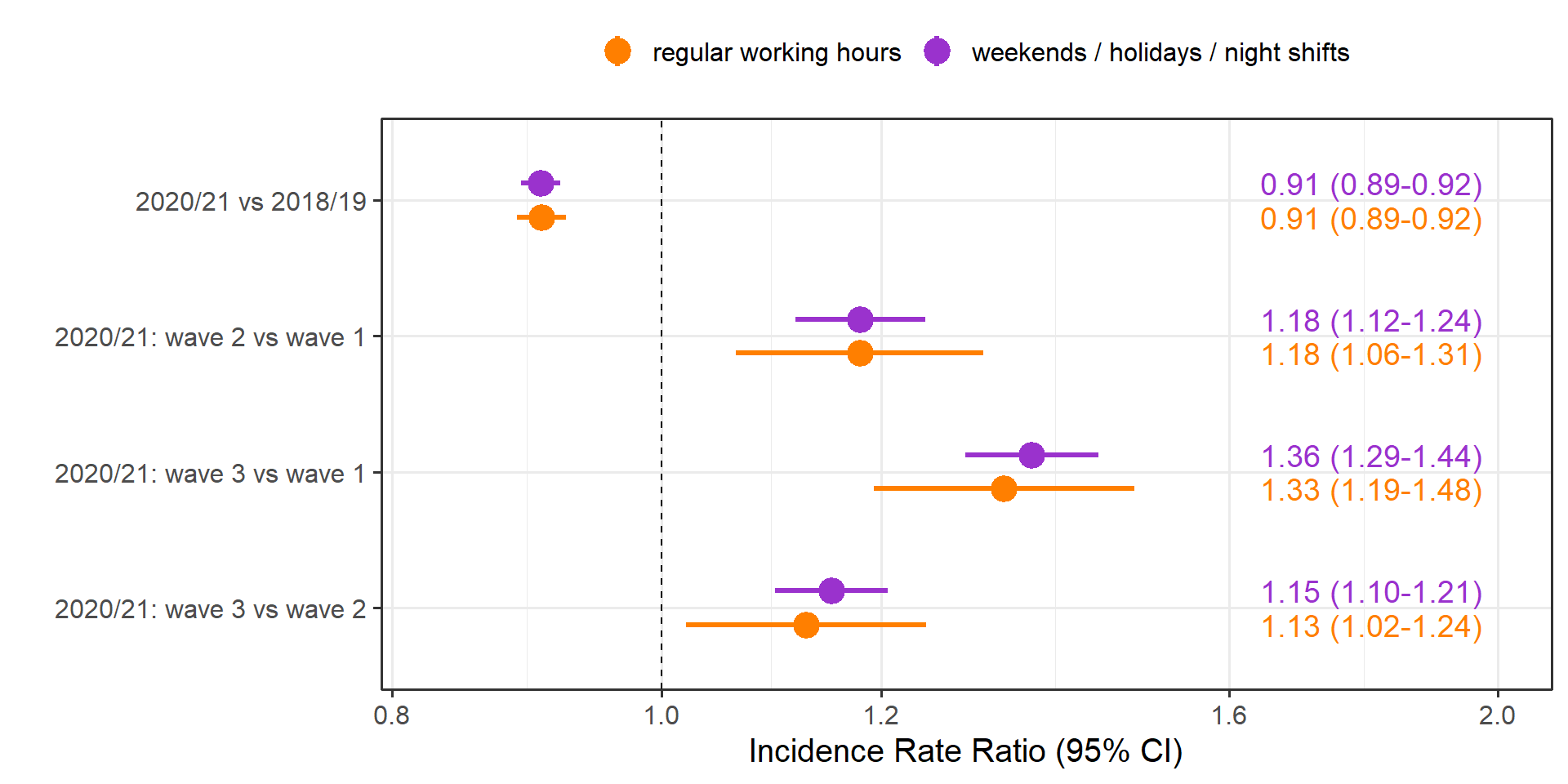
**
